# Decreased Polycystin 2 Levels Result in Non-Renal Cardiac Dysfunction with Aging

**DOI:** 10.1371/journal.pone.0153632

**Published:** 2016-04-15

**Authors:** Ivana Y. Kuo, Sophie L. Duong, Lily Nguyen, Barbara E. Ehrlich

**Affiliations:** 1 Department of Pharmacology, Yale University New Haven, Connecticut, United States of America; 2 Department of Cellular and Molecular Physiology, Yale University, New Haven, Connecticut, United States of America; University of Georgia, UNITED STATES

## Abstract

Mutations in the gene for polycystin 2 (Pkd2) lead to polycystic kidney disease, however the main cause of mortality in humans is cardiac related. We previously showed that 5 month old Pkd2+/- mice have altered calcium-contractile activity in cardiomyocytes, but have preserved cardiac function. Here, we examined 1 and 9 month old Pkd2+/- mice to determine if decreased amounts of functional polycystin 2 leads to impaired cardiac function with aging. We observed changes in calcium handling proteins in 1 month old Pkd2+/- mice, and these changes were exacerbated in 9 month old Pkd2+/- mice. Anatomically, the 9 month old Pkd2+/- mice had thinner left ventricular walls, consistent with dilated cardiomyopathy, and the left ventricular ejection fraction was decreased. Intriguingly, in response to acute isoproterenol stimulation to examine β-adrenergic responses, the 9 month old Pkd2+/- mice exhibited a stronger contractile response, which also coincided with preserved localization of the β2 adrenergic receptor. Importantly, the Pkd2+/- mice did not have any renal impairment. We conclude that the cardiac-related impact of decreased polycystin 2 progresses over time towards cardiac dysfunction and altered adrenergic signaling. These results provide further evidence that polycystin 2 provides a critical function in the heart, independent of renal involvement.

## Introduction

Autosomal Dominant Polycystic Kidney Disease (ADPKD) arises from mutations to either polycystin 1 or 2 (PC1 or PC2, gene name *Pkd1* and *Pkd2*). Patients are born heterozygous, with the renal cysts thought to appear after somatic mutations in the non-mutated allele, in a process called the two-hit hypothesis [1]. Thus, ADPKD is generally a late-onset disorder, with cysts growing large enough to begin causing decreased renal function in the fourth to fifth decade of life. In addition to renal cysts, ADPKD patients also present with a number of extra-renal manifestations, including hypertension and left ventricular cardiac hypertrophy [2, 3]. Despite its name, cardiovascular dysfunction is the main cause of mortality in ADPKD patients. The cardiovascular defects are generally assumed to arise solely due to renal cystic compression of the renal vasculature, leading to hypertension and cardiac dysfunction [4]. Indeed, in the recently completed HALT-PKD human clinical trial that aggressively controlled the blood pressure with a combination therapy of angiotensin converting enzyme inhibitor (ACEi) and angiotensin receptor blocker (ARB), there was decreased development of left ventricular hypertrophy [5, 6].

Intriguingly, a sub-population of ADPKD patients display idiopathic dilated cardiomyopathy before any loss of renal function, showing that ADPKD patients can exhibit cardiac defects before onset of renal impairment [7]. The link between polycystin function and cardiomyopathy is supported by the observation that both polycystins are expressed in the heart, and patients with germ line mutations of the polycystin proteins express dysfunctional or decreased amounts of the polycystin proteins in cells throughout the body, including the heart. Additionally, the cardiac specific *Pkd1* deficient mouse had decreased cardiac function in the absence of renal cysts [8]. This provides strong evidence that expression of aberrant polycystin proteins can act in the heart to compound and give rise to cardiac defects, in addition to their contribution to the formation of renal cysts.

In contrast to PC1, which is primarily found on the plasma membrane and primary cilia, PC2 is found with the primary cilia and extensively in the endoplasmic reticulum (ER) [9, 10]. Within the ER, PC2 can both act as a calcium release channel [11] and as a modulator of other intracellular calcium release channels, namely the inositol tris-phosphate receptor [12, 13], or the ryanodine receptor (RyR) [14]. In cardiomyocytes, PC2 appears to regulate intracellular calcium release via the RyR [7, 15], and thus it is an important modulator of calcium-contraction signaling.

In support of this idea, we previously found that cardiomyocytes isolated from 5 month old (mo) Pkd2+/- mice had increased calcium release upon pacing, but exhibited the same amount of contraction when compared to 5 mo WT mice. This was due to a desensitization of the myofilament to calcium via increased phosphorylation of the myofilament protein, Troponin I [15]. The advantage of utilizing the Pkd2+/- heterozygous mouse is that it best mimics the human germline ADPKD phenotype, in that it has haploinsufficiency of polycystin 2 throughout the animal. However, these mice rarely develop renal cysts, presumably because 5 months is insufficient time to accumulate spontaneous mutations that inactivate the intact copy of Pkd2 in the kidneys. Intriguingly, the Pkd2+/- 5 mo mice did not present with a cardiac phenotype that paralleled either dilated cardiomyopathy or left ventricular hypertrophy, and had preserved ejection fraction [15]. As ADPKD is typically a late-onset disorder in humans, we hypothesized that older Pkd2+/- mice would display evidence of a progressive cardiac phenotype. Here, we find that 9 mo Pkd2+/- mice showed several anatomical features consistent with a dilated cardiac phenotype. Moreover, cardiac function was depressed in the Pkd2+/- 9 mo mice, but the response to adrenergic stimulus was significantly elevated compared with WT controls. Our findings support a model where decreased PC2 levels progressively lead to dilated cardiomyopathy features, similar to human ADPKD patients.

## Materials and Methods

### Animals

All animal husbandry conditions and procedures were conducted according to protocols approved by Yale University IACUC (Protocol #11491). For all studies, only male Pkd2+/- on a C57Bl6 background and WT littermates were used. Mice were either aged 1 mo or 9 mo (as specified in S1 Table). When necessary, wild type (WT) C57Bl6 mice were procured (Jackson Laboratory).

### mRNA and protein studies

Mice were anaesthetized with isoflurane and the left ventricle quickly removed, washed of excess blood in sterile PBS and tissue harvested for either mRNA or protein studies. For mRNA studies, the tissue was snap frozen in liquid nitrogen until required. mRNA was extracted using the RNeasy fibrous tissue kit according to manufacturer’s instructions (Qiagen), reverse transcribed into cDNA using random primers and Multiscribe reagents (Life Technologies). Depending on the gene being amplified, either SYBR green or Taqman primers and reagents were utilized as previously described [15]. For protein studies, tissue was snap frozen, homogenized using a polytron homogenizer in RIPA lysis buffer. After clarifying the supernatant by centrifugation, protein samples were analyzed by SDS-Page followed by wet protein transfer onto PVDF membranes as previously described [15]. Primary antibodies used were PC2 (gift of Dr. Y. Cai and S. Somlo, Yale University), RyR2 (Pierce Antibodies), sarco/endoplasmic reticulum calcium ATPase isoform 2A (SERCA 2A) (Pierce Antibodies), phosphorylated (Ser 16, Santa Cruz) and total forms of phospholamban (Abcam), sodium calcium exchanger (NCX, Santa Cruz), and phosphorylated (Ser 23/24), and total forms of TnI (Cell Signaling Technologies), TnT (Pierce Antibodies) and alpha-tubulin (Abcam).

### Morphological studies

Mice were anaesthetized with isoflurane and perfused through the left ventricle with saline. For histological analysis, the heart and kidney were removed and immersion fixed in 4% formalin, followed by embedding into paraffin wax, and 8 μm sections cut and counterstained with either Masson’s Trichrome or with haematoxylin and eosin. For immunofluorescence analysis, following saline perfusion, the heart was perfused with 2% paraformaldehyde and removed as previously described [15]. After cyro-protection in 30% sucrose, and embedding in optimal cutting temperature media (OCT), 12 μm cryo sections were cut. Sections were blocked in 2% BSA with 0.02% Triton-X in PBS, and then incubated in primary antibodies (both βAR antibodies were obtained from Abcam) overnight at 4°C. After washing, sections were incubated in appropriate secondary antibodies, and mounted in pro-long Gold (Life Technologies). Sections were imaged on a Zeiss 710 confocal microscope using Zen software (Heidelberg, Germany).

### Echocardiography

Mice were anaesthetized with isoflurane through a nose cone and the temperature and respiration rate constantly monitored. After application of a depilating agent, mice were subjected to electrocardiographic analysis using the M-Mode with the short axial measurements of the left ventricle and the Cardiology Package (VEVO 2100, VevoSonics Toronto, Canada) [15].

### Drugs

Isoproterenol, carvedilol and metoprolol were all obtained from Sigma. For control intraperitoneal (IP) injections, either saline or 0.1% DMSO was injected.

### Statistical analysis

Numbers of animals per data set are specified in the legends. For all Western Blot data, each lane represents a separate animal. One-way ANOVA followed by Tukey’s test or Student’s t-test was used to analyze for statistical differences using Prism Software. Data were considered to be significantly different when the p value was less than 0.05.

## Results

### Altered expression of calcium-contractile proteins in 9 mo Pkd2+/- mice

**Fig 1:**
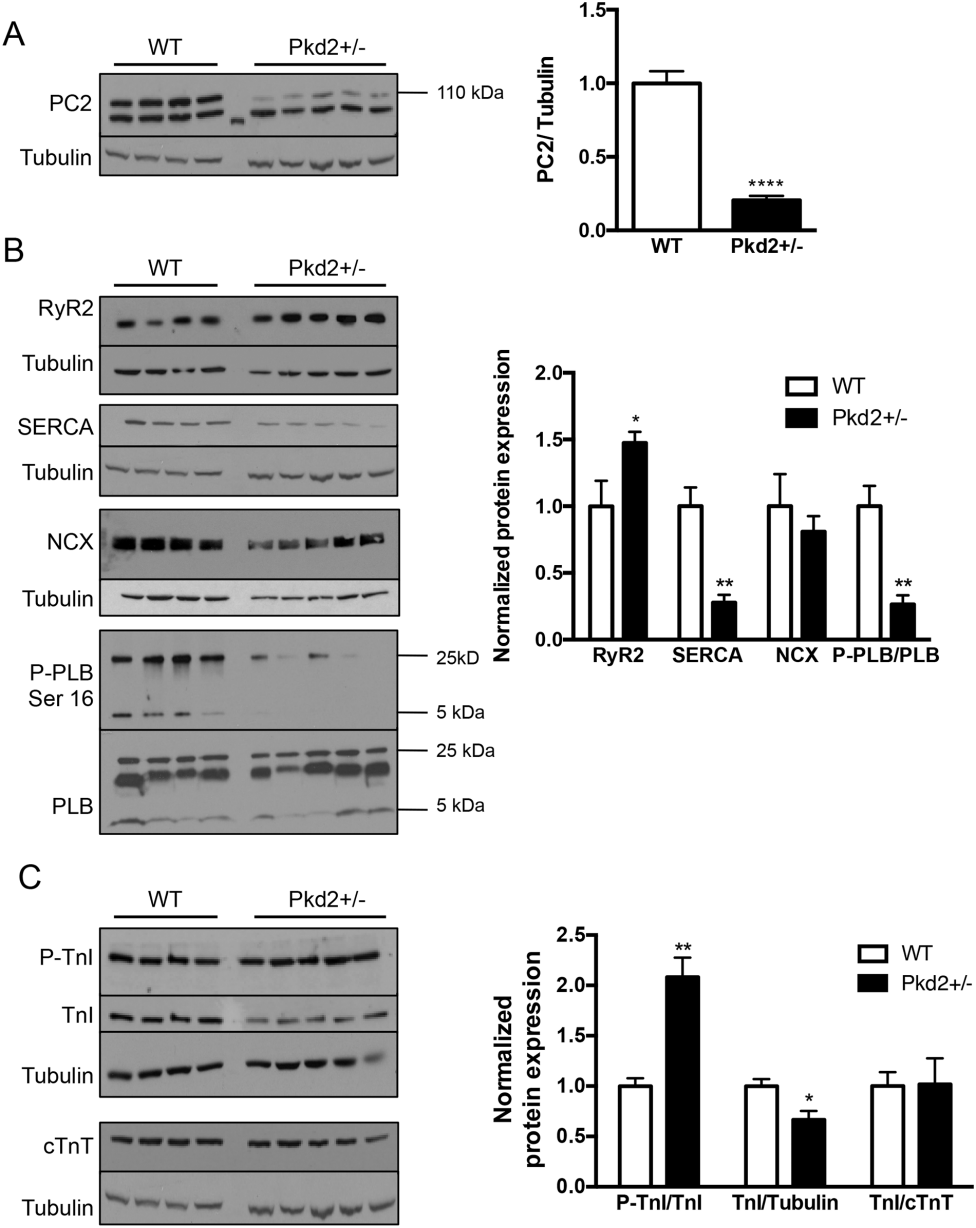
Expression of calcium handling and contractile proteins in 9 mo WT and Pkd2+/- mice. (A) PC2 expression in the left ventricle was diminished by ~70% in Pkd2+/- mice compared with WT mice. (B) Expression of the intracellular calcium release channel RyR2 was increased, whereas SERCA2A and the phosphorylated form of PLB were decreased. NCX levels were unchanged. Data are quantified on the right. RyR2, SERCA2A and NCX are all normalized to tubulin. (C) The expression of the phosphorylated form of TnI was significantly increased in the Pkd2+/- mice. The levels of TnI and cTnT in the Pkd2+/- mice were diminished overall compared to the WT mice. Data are quantified on the right. Each lane represents a separate animal. *p < 0.05, **p < 0.01, ****p<0.0001.

Our studies were conducted on mice of two different ages—1 mo (representing juvenile humans) and 9 mo (representing middle aged humans). The resting heart rate and body weights at both ages between the two genotypes, WT and Pkd2+/-, were indistinguishable (S1 Table). The mRNA expression of the gene was decreased by ~ 50% (S1 Fig), and the protein in 9 mo Pkd2+/- mice was decreased by ~70% compared with WT levels (Fig 1A). The higher molecular weight band at 110 kDa is the specific PC2 band, with the lower weight band considered non-specific. As PC2 is an intracellular calcium channel, and we had previously observed altered calcium handling in the 5 mo Pkd2+/- mice [15], we started by examining the calcium handling proteins in the two different age groups. At 1 mo, there were no significant changes in mRNA expression in Pkd2+/- mice compared to WT controls across a number of genes including Ryanodine Receptor subtype 2 (RyR2), the main intracellular calcium release channel in cardiomyocytes, and sarco/endoplasmic reticulum Ca^2+^ ATPase subtype 2A (SERCA2A), the main intracellular calcium uptake pump (S1A Fig). Analysis of the protein expression of calcium handling proteins revealed a significant decrease in SERCA2A expression, but not in RyR2. There was no change in the phosphorylation levels of phospholamban (PLB), a protein, which when phosphorylated at the Serine 16 site by protein kinase A (PKA), increases uptake of Ca^2+^ via an interaction with SERCA (S1B and S1C Fig).

In the Pkd2+/- 9 mo mice, the calcium handling protein expression profile was very different. RyR2 expression was significantly increased in the Pkd2+/- mice by 50% compared to WT littermates, whereas SERCA2A expression was significantly decreased by 70%. In addition, phosphorylated PLB was significantly decreased in the Pkd2+/- mice compared with WT mice (Fig 1B). However, the total amount of PLB was unchanged. The upregulation of RyR2 and the decreased phosphorylation status of PLB were similar to our findings in 5 mo Pkd2+/- mice [15]. The downregulation of SERCA in the 9 mo mice was of particular interest, as at 5 mo, we had previously observed that the SERCA level was enhanced compared to age-matched controls. The loss in SERCA2A in the Pkd2+/- mice was not compensated by an increase in expression of the plasma membrane localized sodium-calcium exchanger, NCX (Fig 1B). Increased NCX expression is known to be elevated in late-stage heart failure [16, 17].

The bi-phasic change in SERCA expression was not entirely unexpected, as several genes are known to change in the opposite direction between early and late heart failure [18]. One interpretation of the change in SERCA is due to compensation associated with the maintenance of function (ie: the situation at 5 mo) compared with more pathologic changes (the downregulation at 9 mo). Thus, as SERCA downregulation is a well-known marker of cardiac dysfunction [19], we hypothesized that the 9 mo mice would have functional defects consistent with the early signs of heart failure.

We also examined several proteins that are components of the myofilament. In the 9 mo mice, the ratio of the Troponin I (TnI) phosphorylation at Ser 23/24 to total TnI was doubled (Fig 1C). This finding is consistent with our previous observation that TnI phosphorylation was elevated in 5 mo Pkd2+/- mice [15]. Functionally, an increase in phosphorylated TnI would contribute to a decreased sensitivity of the myofilament to intracellular calcium release. Intriguingly, although the ratio of the phosphorylated form of TnI to total TnI was significantly elevated, the total TnI levels were decreased in the 9 mo Pkd2+/- mice when compared with tubulin levels (Fig 1C). To assess if the decreased total TnI expression was due to total myofilament loss, we also examined the total levels of cardiac TroponinT (cTnT). As the stoichiometric levels of cTnT and TnI should be close to 1, it was expected that the ratio between TnI and cTnT would be 1. Indeed, although the levels of cTnT compared to actin was found to be decreased in the Pkd2+/- mice (Fig 1C), the ratio of TnI to cTnT was 1, suggesting that there was a decrease in myofilament proteins overall in the Pkd2+/- 9 mo mice (Fig 1C). However, as the loss of myofiamilament proteins was not replaced by fibrosis, we speculate that the loss of myofilament proteins could be a consequence of the adult forms of TnI and TnT, being replaced with the neonatal forms.

### 9 mo Pkd2+/- mice have signs of dilated cardiomyopathy

**Fig 2:**
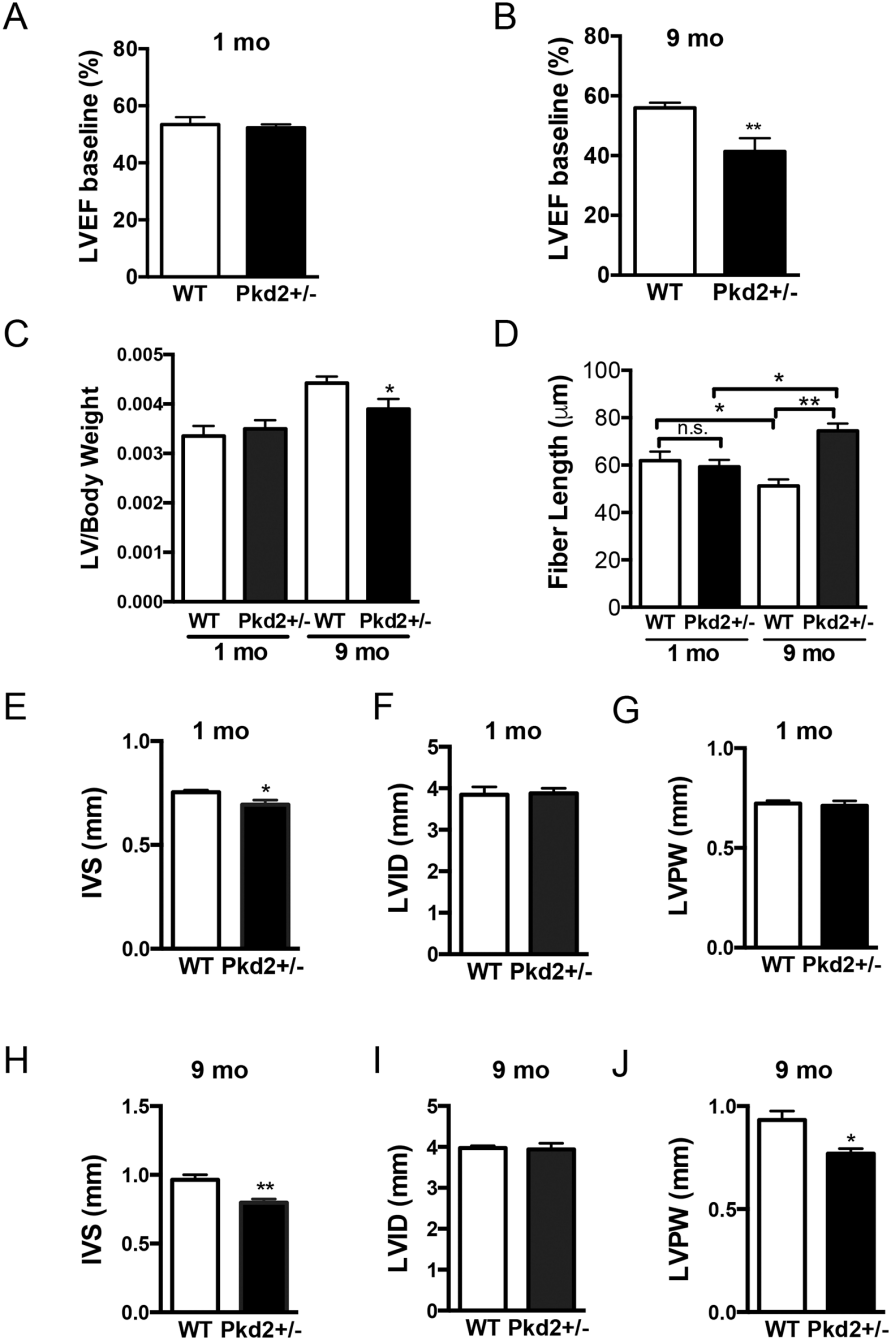
9 mo Pkd2+/- mice have decreased ejection fraction and ventricular remodeling. (A) 1 mo WT and Pkd2+/- mice have similar left ventricular ejection fraction values. (B) 9 mo Pkd2+/- mice have significantly lower left ventricular ejection fraction values compared with WT mice. (C) Left ventricular to body weight ratios were significantly reduced in 9 mo mice but were unchanged in 1 mo mice. (D) Cardiomyocyte lengths were significantly longer in 9 mo mice but are unchanged in 1 mo mice. (E-G) 1 mo Pkd2+/- mice have significantly thinner inner septum measurements compared with WT mice, but the posterior wall and interior diameter are the same. (H-J) 9 mo Pkd2+/- mice have significantly thinner left ventricular wall and inner septum measurements compared with WT mice. Data in A-C, E-J are representative of five 1 mo mice in each group, and eight and nine WT and Pkd2+/- 9 mo mice, respectively. Data in D are representative of at least two different animals per group with 22–45 cells measured from each animal. *p < 0.05, **p < 0.01, n.s. = not significant.

When cardiac function was measured with non-invasive echocardiograms, we observed that the 1 mo Pkd2+/- had no loss in left ventricular ejection fraction (Fig 2A). In contrast, the 9 mo Pkd2+/- mice had a significantly lower left ventricular ejection fraction (58% in WT and 40% in Pkd2+/-, Fig 2B). This suggests that these mice showed signs of decreased cardiac function. Consistent with our finding that SERCA2A levels were decreased, there was also a trend to lower diastolic measurements, but systolic measurements were not significantly different in the 9 mo animals (S2 Table). Not surprisingly, the 1 mo Pkd2 +/- mice had baseline cardiac parameters and measurements that were similar to the WT littermates and within the expected physiological range (S2 Table) [20, 21]. These results demonstrate that the cardiac function of the Pkd2+/- mice is impaired with aging, and that the functional results fit with the biochemical analysis.

As the decreased heart function and the biochemical changes in the 9 mo Pkd2+/- mice are suggestive of cardiomyopathy, we examined the gross anatomical features of the mouse hearts. The LV/body weight ratio was not different at 1 mo, however, the 9 mo Pkd2+/- mice had significantly lower LV/body weight ratios (Fig 2C). In 1 mo animals, the length of the cardiomyocytes collected under diastolic conditions was unchanged between genotypes, however, the 9 mo Pkd2 +/- mice had significantly longer cardiomyocytes compared with the WT counterparts (Fig 2D). In 1 mo mice, the measurements of the heart were essentially similar, although, the inner ventricular septum was thinner in the Pkd2+/- mice (Fig 2E–2G). In comparison, in the 9 mo Pkd2 +/- mice, both the inner septum and the left ventricular posterior wall were significantly thinner than in the WT mice, although the left ventricular inner diameter was unchanged (Fig 2H–2J). These anatomical data are all consistent with a progressive dilated cardiomyopathy phenotype in the 9 mo Pkd2+/- mice. However, as noted above, inconsistent with typical dilated cardiomyopathy, the mass of the left ventricle, was significantly decreased and there was no dilation of the left ventricle, which may suggest that the hearts were displaying an early form of dilated cardiomyopathy.

**Fig 3:**
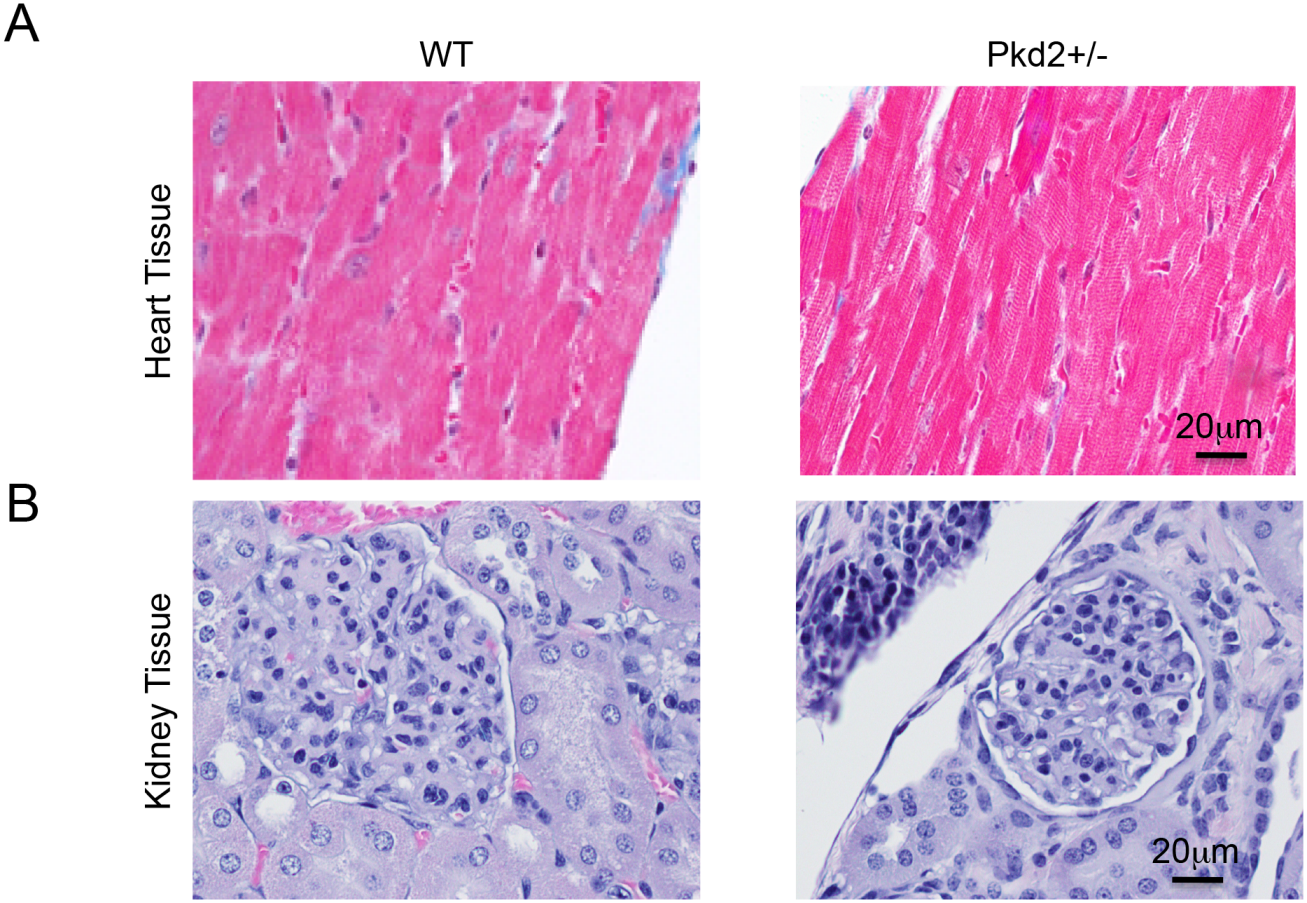
No gross cardiac or renal abnormalities in WT and Pkd2+/- mice. (A) Left ventricles from 9 mo WT (left) and Pkd2+/- (right) mice show similar Masson’s Trichrome staining patterns with no signs of fibrosis. (B) 9 mo WT (left) and Pkd2+/- (right) mice have similar H&E staining patterns with no renal cysts. Data are representative of at least 3 mice in each group.

Masson’s Trichome staining at both 1 and 9 mo revealed no significant deposits of fibrosis, suggesting that the 9 mo Pkd2+/- mice are in the early stages of cardiac dysfunction, before onset of fibrosis (Fig 3A). There was no difference in the Masson’s Trichome staining in the 1 mo Pkd2+/- mice (S2A Fig). Importantly, there was no evidence that the 9 mo Pkd2+/- mice have left ventricular hypertrophy, which would be hypothesized if renal cysts and subsequent hypertension were contributing to the cardiac dysfunction.

To verify that renal cysts were not contributing to the onset of cardiac dysfunction, we examined all mice for renal cysts. None of the 9 mo Pkd2 +/- mice examined had renal cysts, and there was no obvious dilation of the tubules in the kidney of the 9 mo Pkd2+/- (Fig 3B). No cysts were observed in the 1 mo Pkd2+/- mice (S2B Fig). The lack of renal cysts in the Pkd2 +/- mouse model was expected, as the kidneys still have one intact copy of the Pkd2 gene. Thus, our data suggest that the Pkd2+/- mice lose cardiac function in the absence of renal cysts, pointing to a cardiac specific function of PC2.

### 9 mo Pkd2+/- mice have exacerbated adrenergic signaling

**Fig 4:**
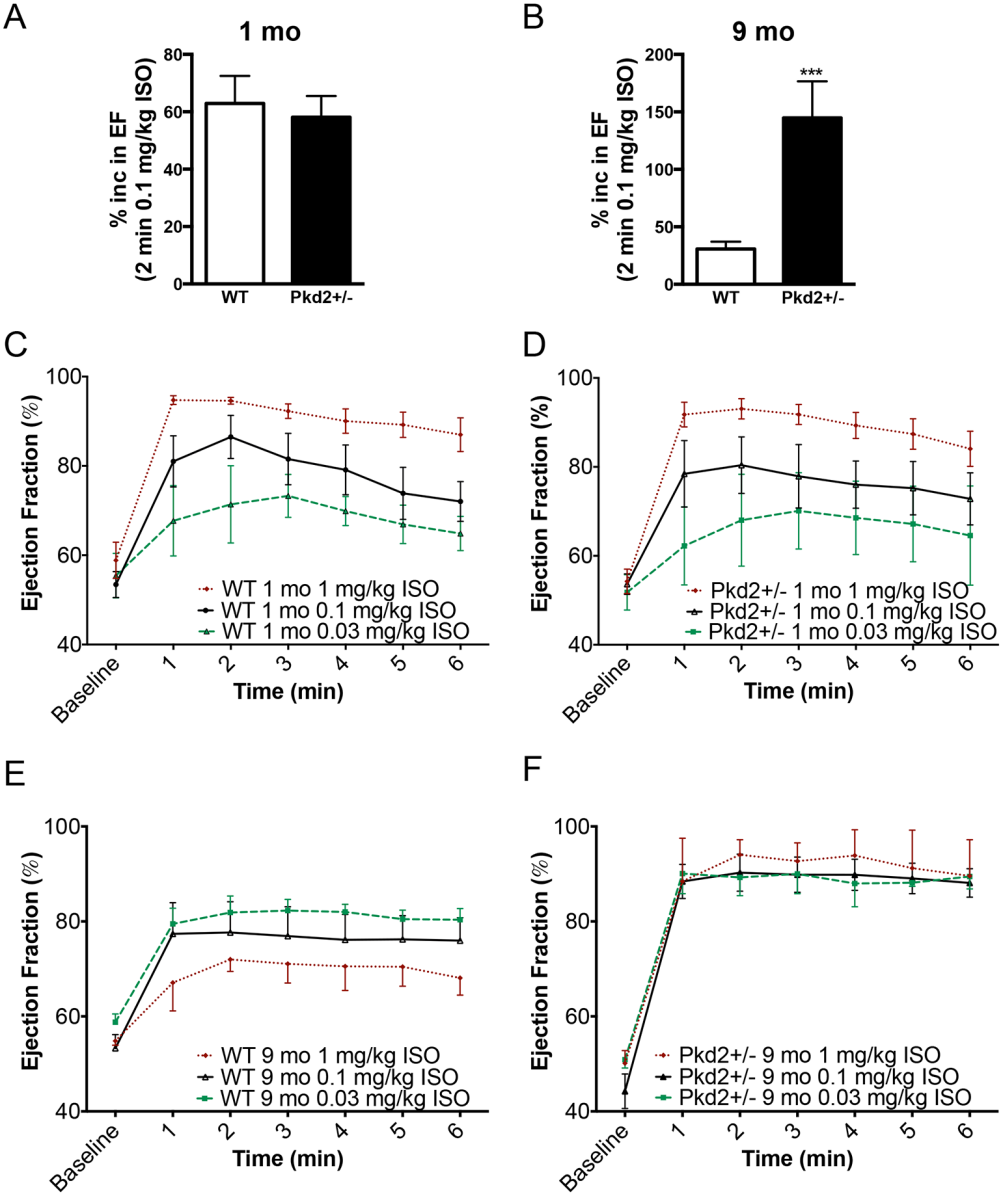
9 mo Pkd2+/- mice have enhanced responses to acute ISO application. (A, B) 1 mo WT and Pkd2+/- mice have comparable left ventricular ejection fraction response 2 min after acute 0.1 mg/kg ISO injection (A) whereas 9 mo Pkd2+/- have a significantly enhanced response (B). (C,D) Using three doses of ISO (0.03, 0.1 and 1 mg/kg), 1 mo WT (C) and Pkd2+/- (D) mice have similar left ventricular ejection fraction responses. (E,F) Using three doses of ISO (0.03, 0.1 and 1 mg/kg), 9 mo WT mice (E) have a dose dependent response, though blunted compared to the 1 mo mice (left). 9 mo Pkd2+/- mice (F) have maximal left ventricular ejection fraction responses to ISO, regardless of the dose. For 1 mo mice, data are representative of 5 mice for each genotype for the 0.1 mg/kg and 1 mg/kg data. For 9 mo mice, the 0.1 mg/kg ISO data are representative of 8 and 9 WT and Pkd2+/-, respectively. For the 0.03 mg/kg ISO data, the 1 mo and 9 mo data are representative of 3 mice. The 1 mg/kg ISO data for 9 mo mice are representative of 3 mice per group.

We had previously found that the 5 mo Pkd2+/- had altered sensitivity to the specific β-adrenergic receptor agonist, isoproterenol (ISO)[15]. To determine if the decreased cardiac function in the 9 mo Pkd2+/- was coupled to a decrease in adrenergic responses, we administered acute intraperitoneal injections of ISO to the mice. Taking a single time point, 2 min after an ISO injection (0.1 mg/kg), there was no significant change between the left ventricular ejection fraction in WT and Pkd2+/- mice at 1 mo (Fig 4A). However, in the 9 mo mice, there was an 80% increase in ejection fractions in the Pkd2+/- compared with the WT mice, demonstrating that the Pkd2+/- mice had a heightened contractile response to ISO (Fig 4B). To examine this phenomenon more carefully, we compared 3 different doses of ISO. At 1 mo, both the WT and the Pkd2+/- mice exhibited a dose dependent increase (0.03, 0.1 and 1 mg/kg ISO) in ejection fraction that was not significantly different from each other over the analyzed 6 min post injection period (Fig 4C and 4D, S3 Fig). In striking contrast, the 9 mo Pkd2+/- mice had a maximal increase in ejection fraction at all three doses (greater than 90%), whereas the 9 mo WT mice had a suppressed ISO response (around 70%) over a 6 min post-injection period (Fig 4E and 4F). The results seen in the WT mice are consistent with the expected decreased adrenergic response in aging rodents [22]. Both the systolic as well as the diastolic measurements were significantly altered in the 9 mo Pkd2+/- mice compared with WT mice (S4 Fig).

**Fig 5:**
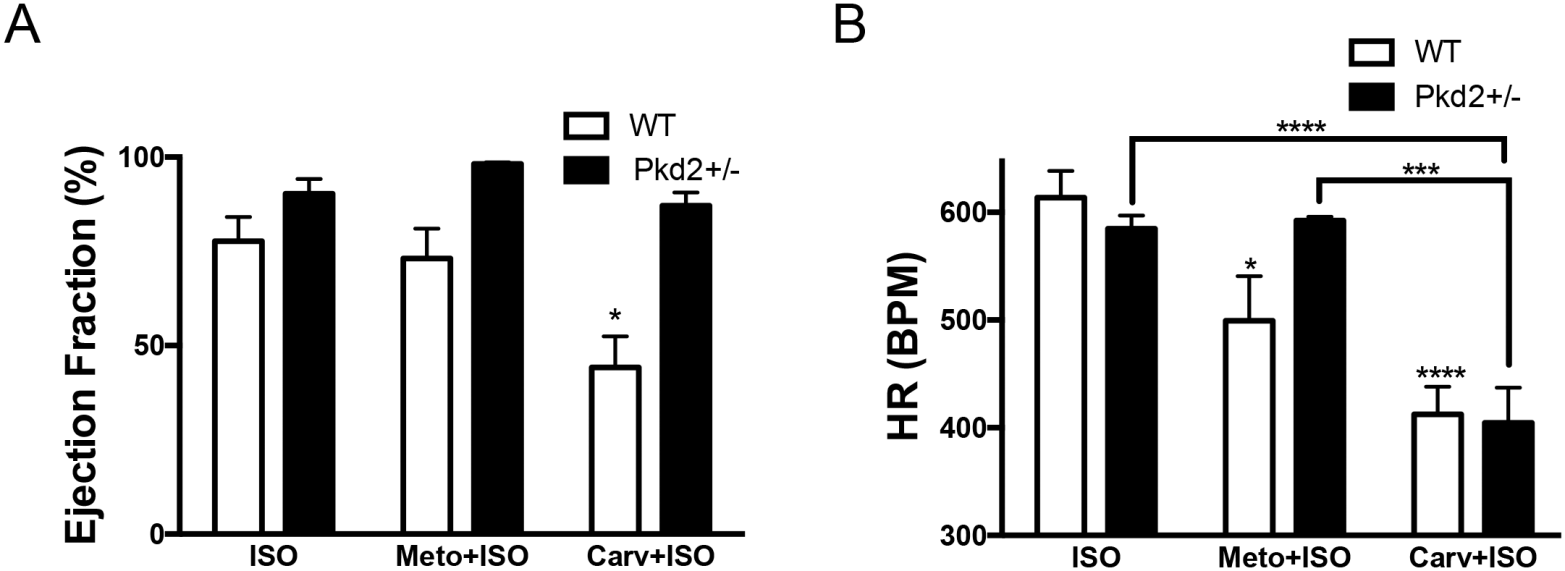
9 mo Pkd2+/- mice have blunted responses to β-blockers. (A) Pre-treating WT and Pkd2+/- mice with the βAR-1 blocker metoprolol does not alter the ejection fraction after ISO challenge, whereas pre-treatment with the pan β-blocker carvedilol diminishes the WT response but not the Pkd2+/- response. (B) The heart rate was significantly decreased after either metoprolol or carvedilol pre-treatment followed by ISO challenge in WT mice, compared to ISO only challenge. In Pkd2+/- mice, only carvedilol, not metoprolol has an effect on heart rate. Note that heart rate after carvedilol pre-treatment is the not significantly different in WT and Pkd2+/- mice. The data are representative of 3–4 mice per group.

Our results after administration of ISO suggested that the 9 mo Pkd2+/- mice have an elevated βAR agonist response, whereas the decrease in ISO response in the 9 mo WT mice has been ascribed to a reduction in βAR-1 expression with aging, resulting in a diminished response to ISO [23]. To determine if the increased ISO response in the 9 mo Pkd2+/- mice was due to an upregulation of the βAR-1 or βAR-2 pathway, animals were injected with β-blockers 30 min prior to ISO challenge. Although β-blockers are known to improve cardiac function over time, acute administration of β-blockers depresses function. For 9 mo mice, we used metoprolol (an antagonist that preferentially targets βAR-1) and carvedilol (a pan βAR blocker that inhibits βAR-1 and βAR-2 with similar efficacy). Metoprolol was selected because it was used as a third line treatment in the HALT PKD human trials [24]. In WT and Pkd2+/- mice, pretreatment with metoprolol did not significantly alter the ejection fraction after ISO challenge, although metoprolol significantly reduced the heart rate in the WT mice. With carvedilol pretreatment, the heart rate was reduced by the same amount in both the WT and Pkd2+/- mice, but intriguingly, whereas there was a significantly diminished ejection fraction in the WT mice, there was no diminution in ejection fraction observed in the Pkd2+/- mice (Fig 5 and S5 Fig). Our observation that carvedilol pretreatment in the 9 mo Pkd2+/- mice had no effect on ejection fraction after ISO challenge further supports the idea that there is an alteration in the adrenergic signaling pathway in the Pkd2+/- mice.

### Expression change of βAR-1 and 2

**Fig 6:**
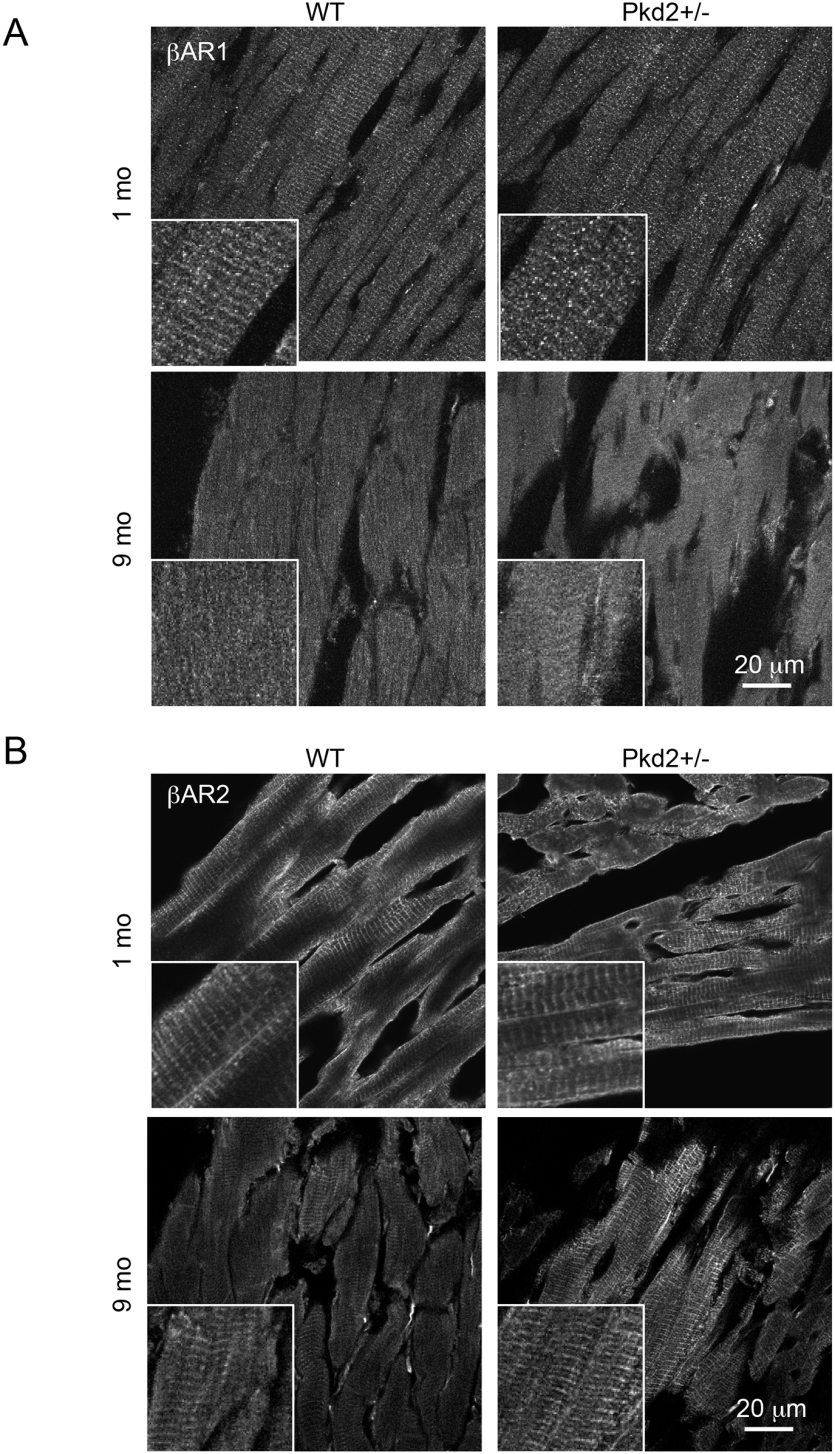
β-Adrenergic receptor expression is altered in 9 mo Pkd2+/- mice. (A) Staining intensity for βAR-1 was similar in 1 and 9 mo WT and Pkd2+/- mice. (B) Staining intensity for βAR-2 was similar in 1 mo WT and Pkd2+/- mice, but increased in 9 mo Pkd2+/- mice, and had a more striated pattern in the Pkd2+/- mice. Insets represent higher magnification images. Images are representative of staining taken from at least 3 separate mice.

To complement our pharmacological approach, we also examined the protein expression and distribution of βAR-1 and βAR-2 in heart sections by Western blot analysis and immunohistochemistry. Analysis of total expression by Western Blot did not demonstrate any significant change in expression between the WT and Pkd2+/- mice at 9 mo (S6 Fig). We then turned to immunohistochemistry to determine if there was a change in the distribution of the βAR, as it has been previously shown that the localization of the βAR (i.e.: associated with T-tubule or plasma membrane), rather than overall expression, is more important in the receptor’s ability to couple to down-stream targets [22]. The staining patterns of both βAR-1 and 2 followed the T-tubule distribution on the myofibrils at 1 mo in both WT and Pkd2+/- mice (Fig 6). However, the staining of βAR-1 pattern was largely lost in both the WT and the Pkd2+/- mice at 9 mo, although the overall expression level of βAR-1 was found to be unchanged between the two groups. In contrast, the βAR-2 staining pattern was maintained in the 9 mo Pkd2+/- mice whereas the striated pattern of staining was lost in the 9 mo WT mice, consistent with previously reported data [22]. These data show that the βAR-2 signaling pathway is upregulated in the Pkd2+/- mice with aging.

## Discussion

**Fig 7:**
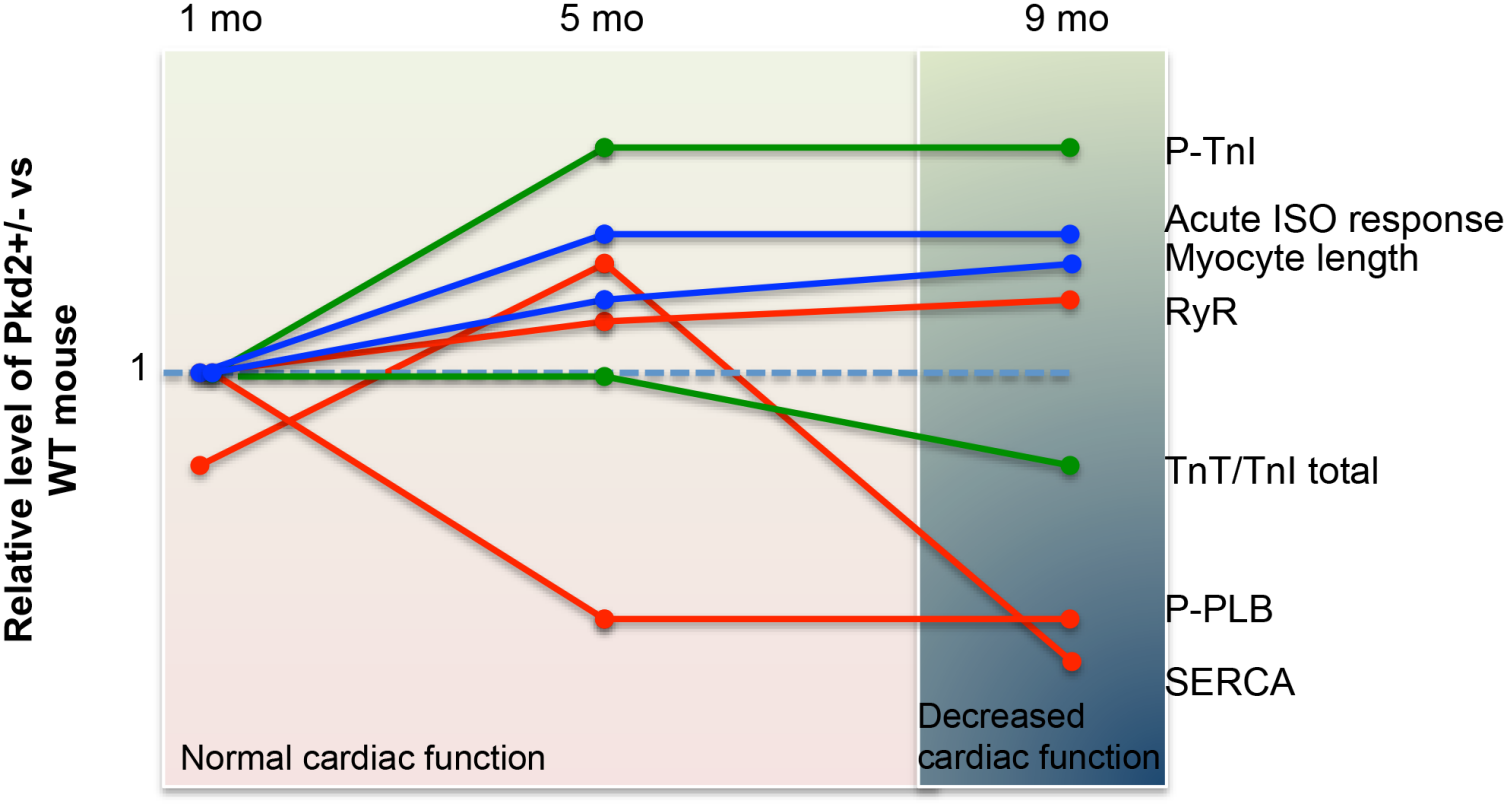
Summary of the changes in protein expression and cardiac function in the Pkd2+/- mice examined over a 9 mo period. Data is summarized from the current paper as well as data from 5 mo mice [15]. Each line represents the expression levels of various proteins (SERCA, RyR, p-TnI, TnI, p-PLB) or other functional readouts (eg: cardiomyocyte length, acute response to ISO) at 1, 5 and 9 mo of age. The orange lines represent those associated with the calcium signaling apparatus (SERCA, RyR, PLB). The green lines represents those proteins associated with the contractile apparatus (p-TnI and cTnT). The blue lines represent other measurements (cardiomyocyte cell length and response to acute ISO stimulus). The background shading (pink and blue) represents the overall cardiac function of the mouse, as measured with echocardiogram.

We demonstrate that Pkd2+/- mice have cardiac abnormalities that become more pronounced with aging and mimic the human cardiac defects that arise independent of renal cyst development. Taking the present data with our previous findings [15], we propose that decreased PC2 in mice is associated with the slow progression of dilated cardiomyopathy, where the heart undergoes several compensatory steps before reaching the stage of cardiac dysfunction at 9 mo of age (Fig 7). These compensatory steps include changes in expression of proteins such as SERCA2A in a bi-phasic manner over the time that we analyzed the mice. In contrast, other protein changes are more consistent between 5 and 9 mo, such as the phosphorylation statuses of both TnI and PLB (Fig 7).

Importantly, we find that the Pkd2+/- mice mimic a phenotype that has more similarities to dilated cardiomyopathy than hypertrophic cardiac myopathy. Although it is expected that human ADPKD patients with renal cysts would display hypertrophic cardiomyopathy, which would be associated with pressure overload, it is less expected to assume that renal hypertension would lead to dilated cardiomyopathy. Our data therefore suggests that the polycystins should also be considered as a gene that modifies or induces the incidence of idiopathic dilated cardiomyopathy. Unlike hypertrophic cardiomyopathy, a greater proportion of patients have idiopathic dilated cardiomyopathy, where the causative gene is unknown [25]. Over the past few years, several new proteins have been implicated as causative of idiopathic dilated cardiomyopathy [26, 27] and the results presented here show that Pkd2 should be added to the list.

Moreover, we find evidence that the Pkd2+/- mice undergo adrenergic remodeling that is sustained between the 5 and 9 mo time points. Although these changes may initially act to compensate the alterations in calcium handling and maintain cardiac contractility (as seen in the 5 mo old mice), ultimately, the preference to βAR-2 may have underlying pathological ramifications. Changes in the adrenergic signaling cascade have been observed in heart failure, and treatment with non-specific β-blockers have been partially successfully in rodent models displaying dilated cardiomyopathy [28], as well as in human trials [29]. Thus, future studies to determine whether β-blockers, and, more specifically, βAR-2 blockers are effective in reversing the progression to cardiac failure in ADPKD patients are warranted.

As catecholamines such as noradrenaline are known to be elevated in ADPKD patients [30], it is possible that the alpha-adrenergic pathway may also be elevated. However, given the specificity of ISO for the βAR pathway, it is unlikely that the changes that we are observing are due to activation of this pathway. Future studies will be required to investigate this possibility.

## Conclusion

Collectively, our data are consistent with the finding that human ADPKD patients can exhibit IDCM before loss of renal function, and provide strong evidence that the polycystins function in the heart to modulate cardiac dysfunction. Moreover, as the adrenergic pathway is elevated with aging, this pathway may present a future therapeutic opportunity.

## Supporting Information

S1 Fig Expression of calcium-contractile proteins in 1 mo old mice. (A) mRNA expression of calcium-contractile genes in 1 mo WT and Pkd2+/- mice. (B) Protein expression of calcium-contractile proteins in 1 mo WT and Pkd2+/- mice. Tissue was taken from the LV (Left ventricle). Each lane is a separate animal. (C) Quantification of samples from panel B normalized to tubulin, or PLB (for p-PLB).
(TIF)

S2 Fig Morphology of 1 mo old mice. (A) Whole hearts from 1 mo WT (left) and Pkd2+/- (right) mice show similar Masson’s Trichrome staining patterns with no signs of fibrosis. (B) 1 mo WT (left) and Pkd2+/- (right) mice have similar H&E staining patterns with no renal cysts. Data are representative of at least 3 mice in each group.
(TIF)

S3 Fig Echocardiographic response of 1 mo old mice to ISO. 1 mo WT (left) and Pkd2+/- (right) cardiac responses to varying doses of ISO over a 6 min period. Y-axes on each graph denotes the parameter being measured. Data are representative of 5 mice for each genotype for the 0.1 mg/kg and 1 mg/kg groups, and 3 mice for the 0.03 mg/kg group.
(TIF)

S4 Fig Echocardiographic response of 9 mo old mice to ISO. 9 mo WT (left) and Pkd2+/- (right) cardiac responses to varying doses of ISO over a 6 min period. Y-axes on each graph denotes the parameter being measured. The 0.1 mg/kg ISO data are representative of 8 and 9 WT and Pkd2+/- mice respectively. The 0.03 mg/kg and 1 mg/kg ISO data are representative of 3 mice for each genotype.
(TIF)

S5 Fig Echocardiographic response to β-blockers. Cardiac responses of 9 mo WT (black traces) and Pkd2+/- (red traces) mice to either pre-treatment with βAR-1 blocker metoprolol (Meto) followed by ISO challenge (top 4 panels) or the pan β-blocker carvedilol (Carv, bottom 4 panels). The data are representative of 3–4 mice per group.
(TIF)

S6 Fig Expression of βAR. Protein expression of βAR-1 (top) and βAR-2 (bottom) as measured by Western Blot analysis. No significant difference was found in the global expression of the two proteins. Note that the tubulin control for βAR-2 is the same data as used for the NCX blot in Fig 1.
(TIF)

S1 Table Age, number, weight and baseline HR of WT and Pkd2+/- mice used for the echocardiograms measurements in this study. Values are Mean (SEM).
(DOCX)

S2 Table Baseline values for WT and Pkd2+/- mice. Values are Mean (SEM).
(DOCX)
