# Supplementary figures and images for: Decreased Polycystin 2 Levels Result in Non-Renal Cardiac Dysfunction with Aging
Source: PLoS One. 2016 Apr 15;11(4):e0153632. doi: 10.1371/journal.pone.0153632 (PMC4833351; doi:10.1371/journal.pone.0153632)

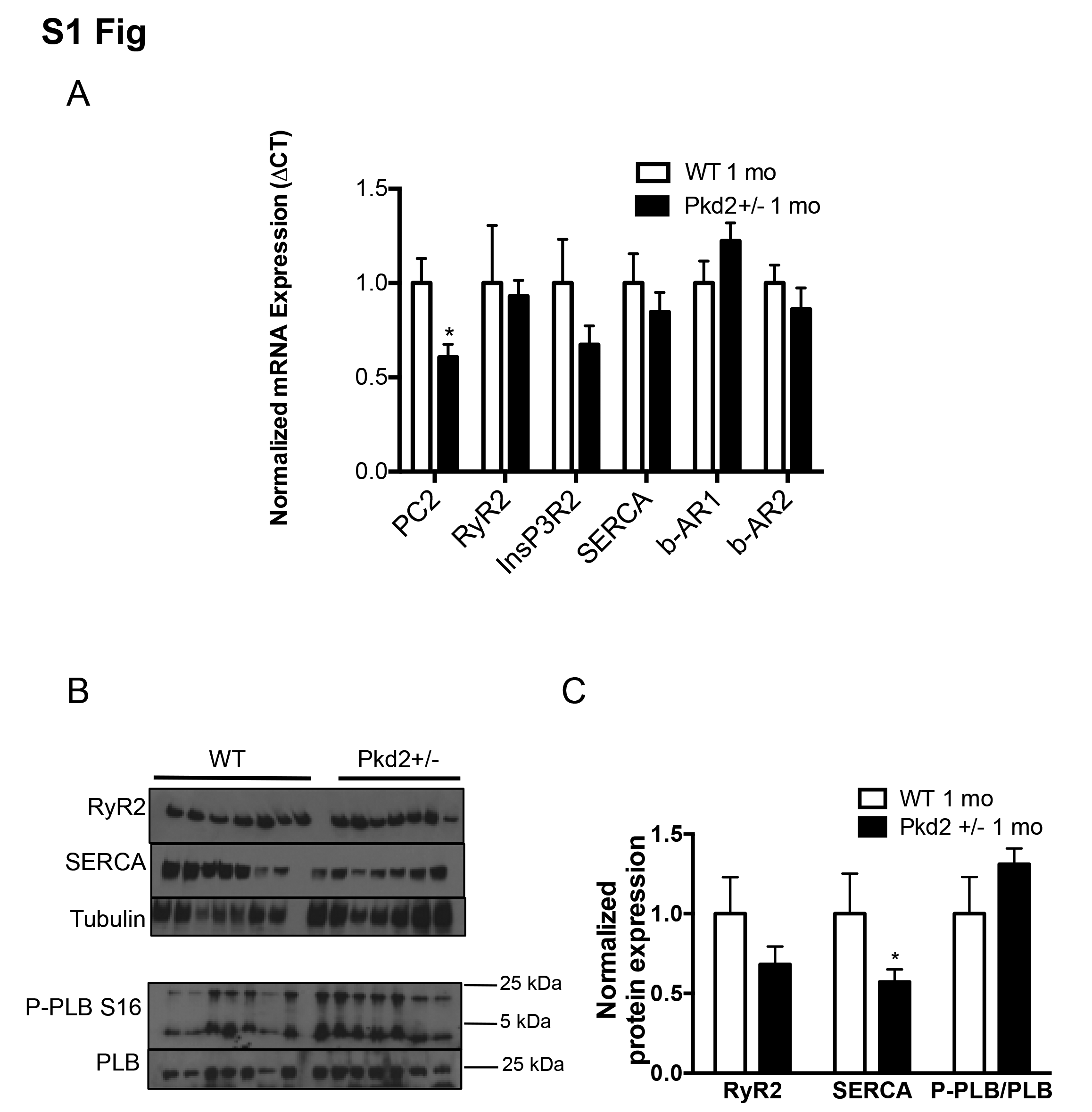

Supplement: S1 Fig — (A) mRNA expression of calcium-contractile genes in 1 mo WT and Pkd2+/- mice. (B) Protein expression of calcium-contractile proteins in 1 mo WT and Pkd2+/- mice. Tissue was taken from the LV (Left ventricle). Each lane is a separate animal. (C) Quantification of samples from panel B normalized to tubulin, or PLB (for p-PLB). (TIF) [file pone.0153632.s001.tif]

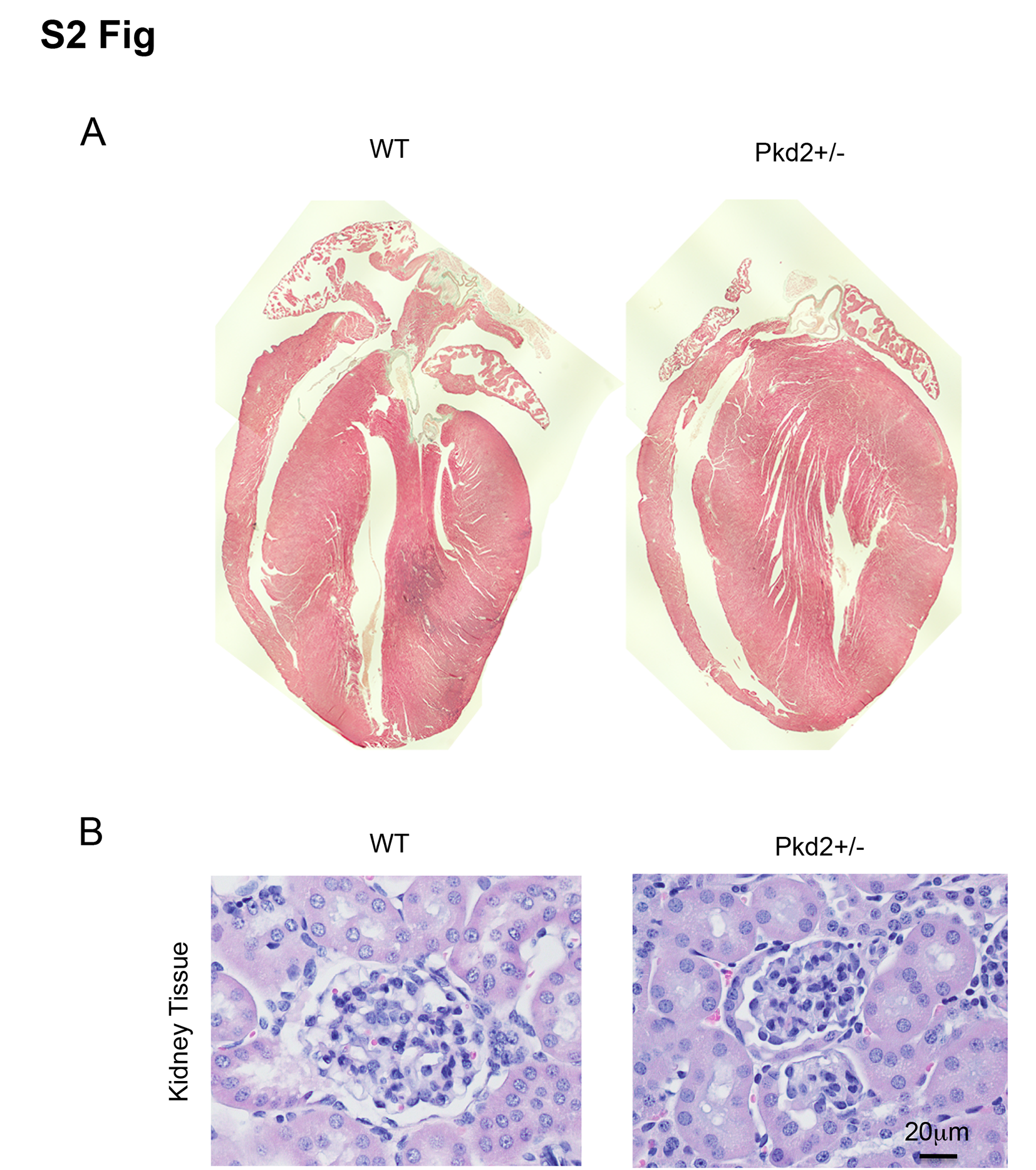

Supplement: S2 Fig — (A) Whole hearts from 1 mo WT (left) and Pkd2+/- (right) mice show similar Masson’s Trichrome staining patterns with no signs of fibrosis. (B) 1 mo WT (left) and Pkd2+/- (right) mice have similar H&E staining patterns with no renal cysts. Data are representative of at least 3 mice in each group. (TIF) [file pone.0153632.s002.tif]

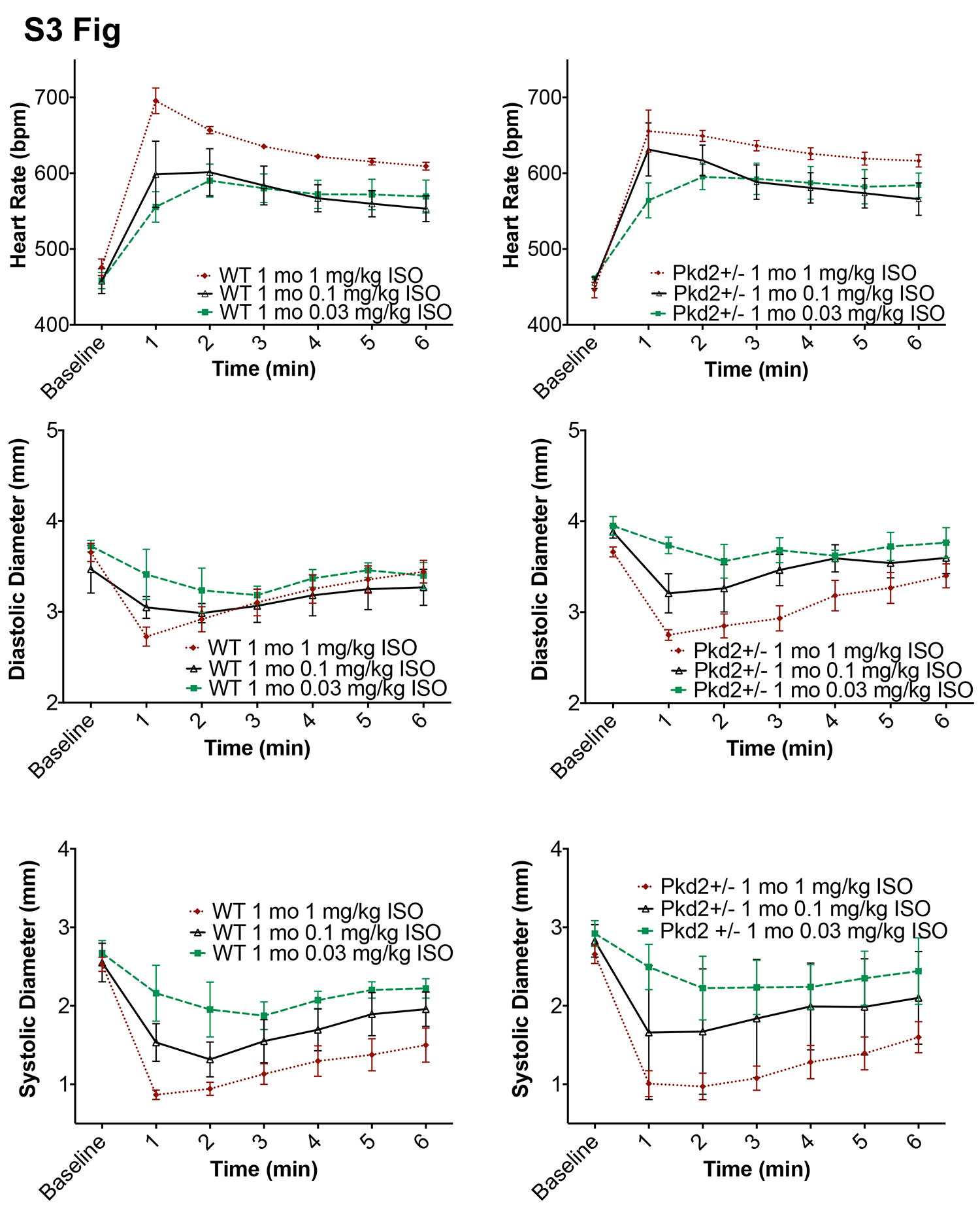

Supplement: S3 Fig — 1 mo WT (left) and Pkd2+/- (right) cardiac responses to varying doses of ISO over a 6 min period. Y-axes on each graph denotes the parameter being measured. Data are representative of 5 mice for each genotype for the 0.1 mg/kg and 1 mg/kg groups, and 3 mice for the 0.03 mg/kg group. (TIF) [file pone.0153632.s003.tif]

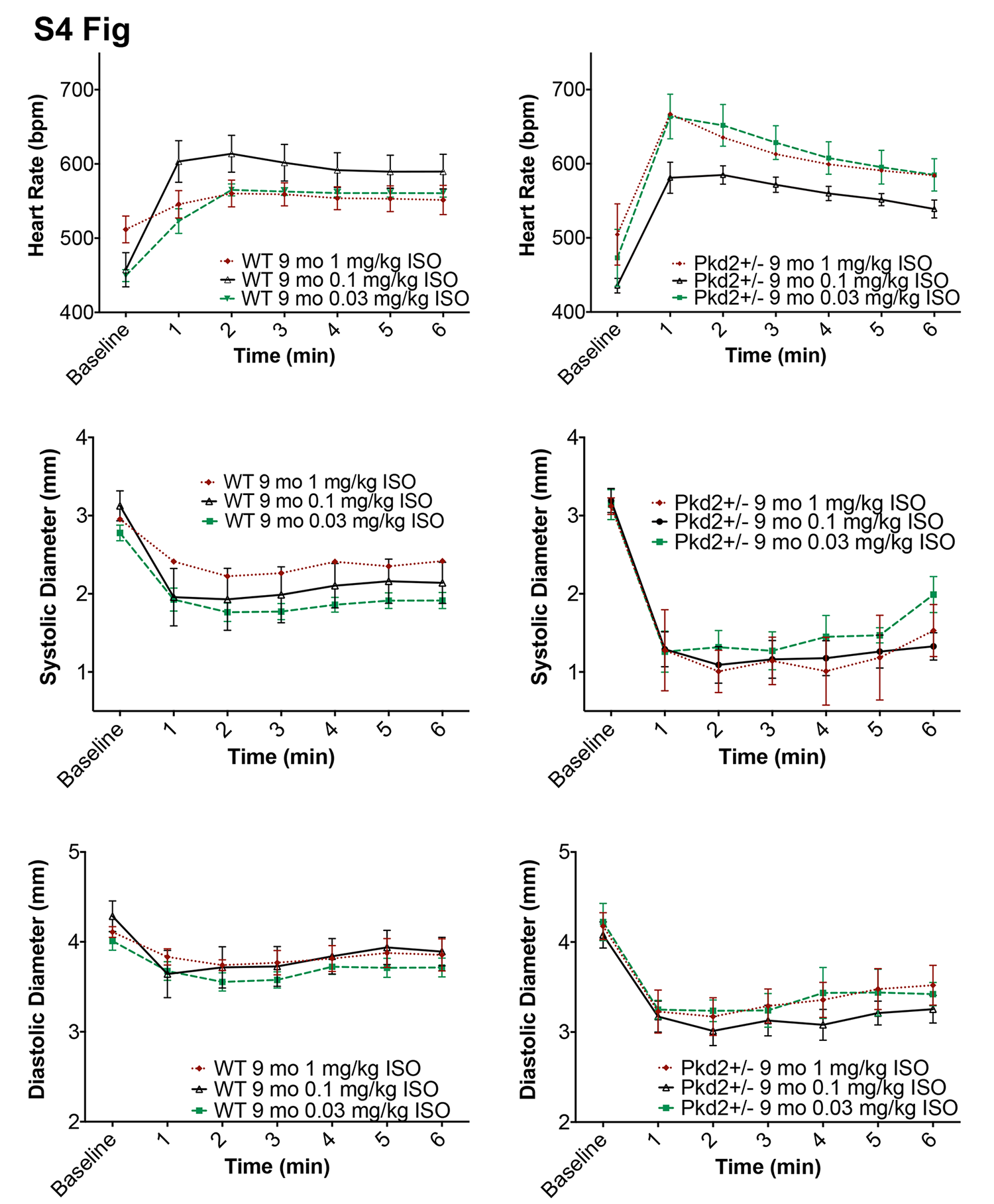

Supplement: S4 Fig — 9 mo WT (left) and Pkd2+/- (right) cardiac responses to varying doses of ISO over a 6 min period. Y-axes on each graph denotes the parameter being measured. The 0.1 mg/kg ISO data are representative of 8 and 9 WT and Pkd2+/- mice respectively. The 0.03 mg/kg and 1 mg/kg ISO data are representative of 3 mice for each genotype. (TIF) [file pone.0153632.s004.tif]

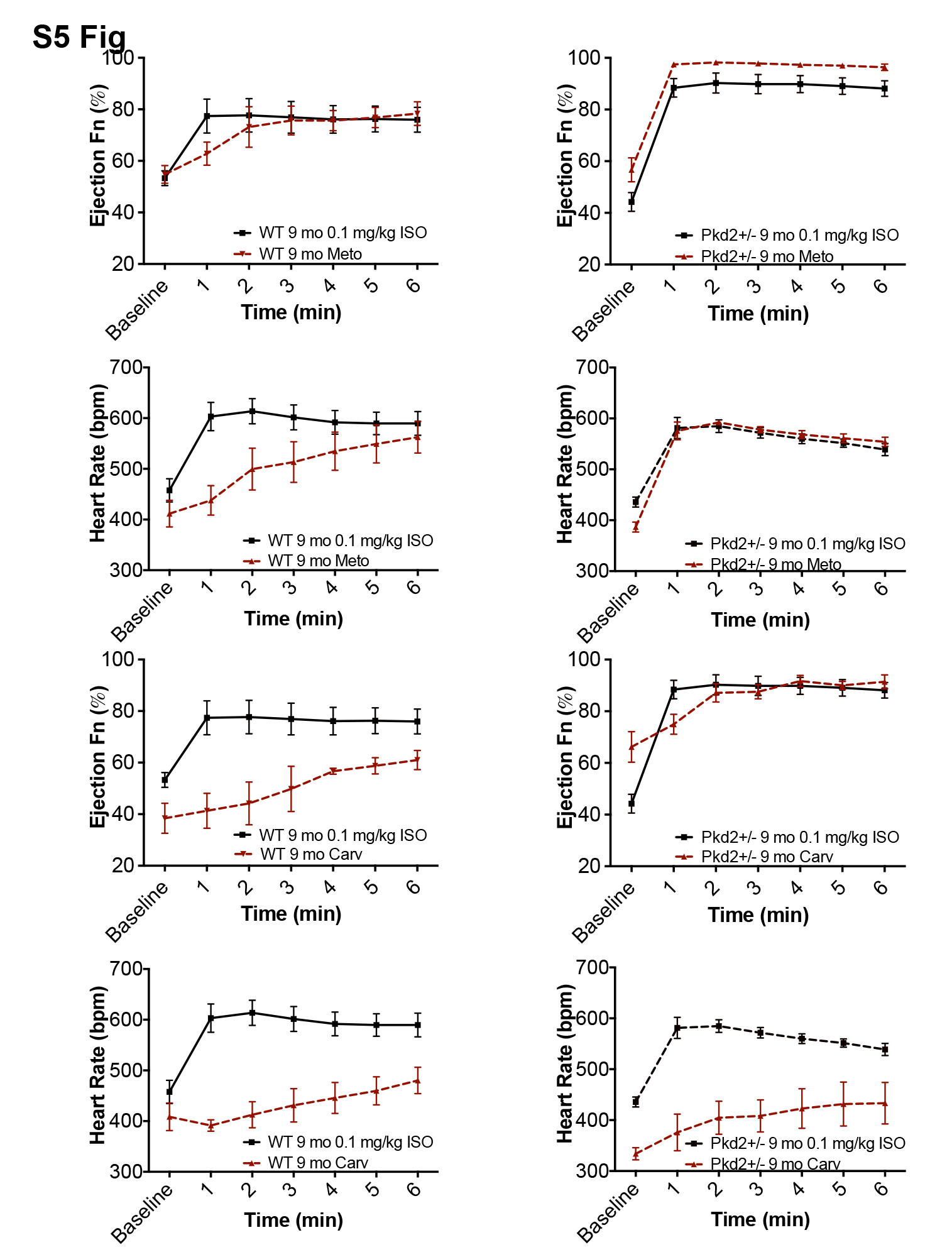

Supplement: S5 Fig — Cardiac responses of 9 mo WT (black traces) and Pkd2+/- (red traces) mice to either pre-treatment with βAR-1 blocker metoprolol (Meto) followed by ISO challenge (top 4 panels) or the pan β-blocker carvedilol (Carv, bottom 4 panels). The data are representative of 3–4 mice per group. (TIF) [file pone.0153632.s005.tif]

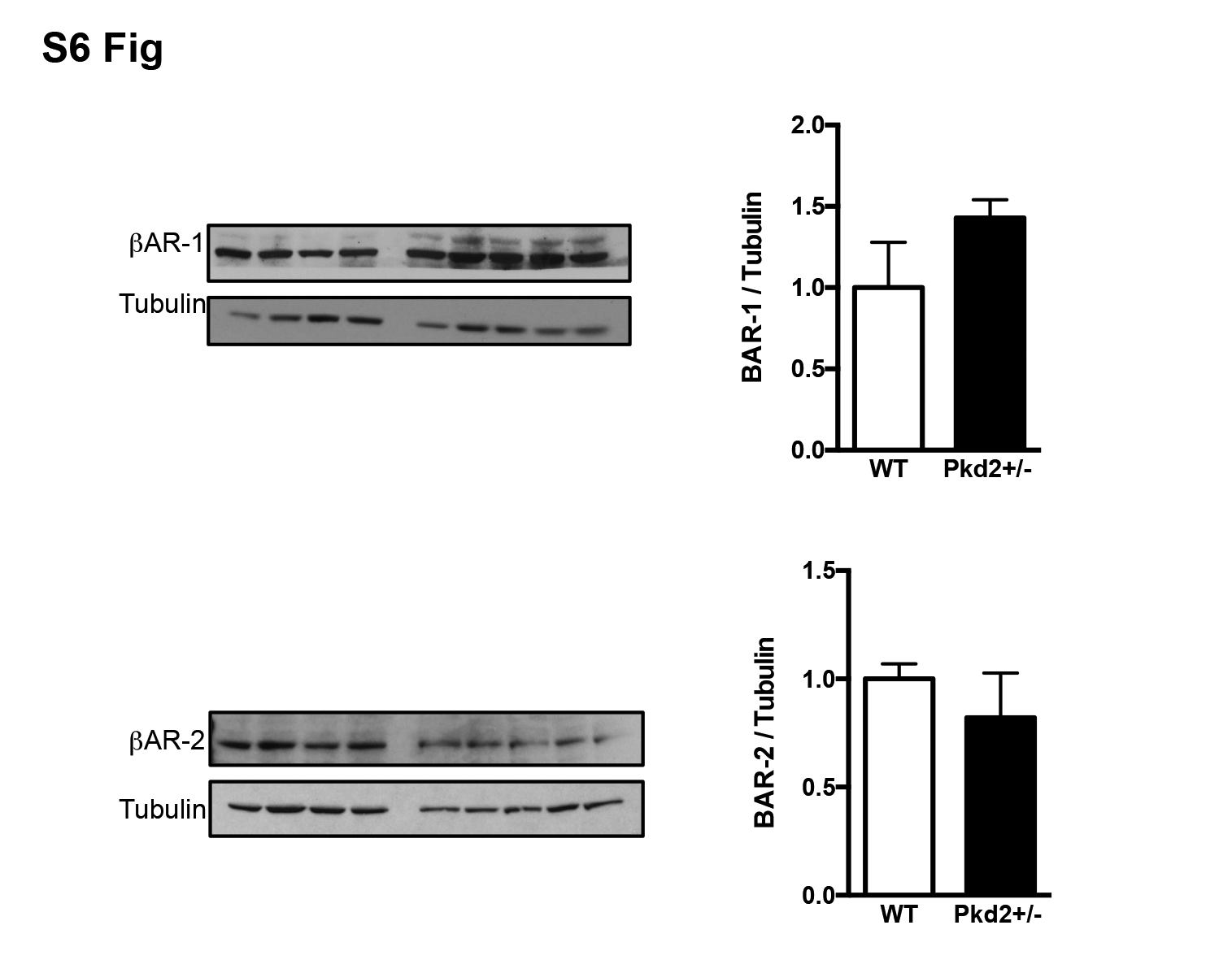

Supplement: S6 Fig — Protein expression of βAR-1 (top) and βAR-2 (bottom) as measured by Western Blot analysis. No significant difference was found in the global expression of the two proteins. Note that the tubulin control for βAR-2 is the same data as used for the NCX blot in Fig 1. (TIF) [file pone.0153632.s006.tif]
