# Supplementary material for: Decreased Polycystin 2 Levels Result in Non-Renal Cardiac Dysfunction with Aging
Source: PLoS One. 2016 Apr 15;11(4):e0153632. doi: 10.1371/journal.pone.0153632 (PMC4833351; doi:10.1371/journal.pone.0153632)
Supplement: S1 Table — Values are Mean (SEM). (DOCX) [file pone.0153632.s007.docx]

**S1 Table.** **Age, number, weight and baseline HR of WT and Pkd2+/- mice used for the echocardiograms measurements in this study.** Values are Mean (SEM).

| Group | N | Weeks (months) | Weight (SEM) | Baseline HR under anaesthesia (SEM) |
| --- | --- | --- | --- | --- |
| Pkd2+/- | 5 | 4-5 (1) | 21.4 (0.5) | 455 (7.5) |
| WT | 5 | 4-5 (1) | 19.9 (1.3) | 457 (16.4) |
| Pkd2+/- | 6 | 35-38 (9) | 31.1 (2.8) | 430.6 (10.5) |
| WT | 10 | 35-39 (9) | 35.1 (1.4) | 468.8 (21.6) |
