# Supplementary material for: Decreased Polycystin 2 Levels Result in Non-Renal Cardiac Dysfunction with Aging
Source: PLoS One. 2016 Apr 15;11(4):e0153632. doi: 10.1371/journal.pone.0153632 (PMC4833351; doi:10.1371/journal.pone.0153632)
Supplement: S2 Table — Values are Mean (SEM). (DOCX) [file pone.0153632.s008.docx]

**S2 Table**. **Baseline values for WT and Pkd2+/- mice.** Values are Mean (SEM).

|  | **WT 1 month** | **Pkd2+/- 1 month** | **WT 9 month** | **Pkd2+/- 9 month** |
| --- | --- | --- | --- | --- |
| Heart Rate | 457.7  (14.7) | 455.5  (6.8) | 468.8  (21.6) | 429.5  (13.3) |
| Systolic Diameter | 2.55  (0.22) | 2.83  (0.08) | 2.96  (0.19) | 3.19  (0.15) |
| Diastolic Diameter | 3.47  (0.24) | 3.88  (0.06) | 4.14  (0.17) | 4.08  (0.14) |
| Systolic Volume | 24.67  (4.88) | 30.43  (2.24) | 34.75  (5.71) | 41.85  (4.65) |
| Diastolic Volume | 51.30  (7.86) | 65.40  (2.46) | 76.75  (7.32) | 74.35  (6.19) |
| Stroke Volume | 26.62  (3.05) | 34.98  (1.35) | 42.00  (2.67) | 32.50  (3.16) |
| Ejection Fraction | 53.44  (2.59) | 53.63  (2.07) | 55.65  (3.11) | 44.24  (3.62) |
| Fractional Shortening | 26.88  (1.49) | 27.26  (1.32) | 28.81  (1.92) | 21.84  (2.06) |
| Cardiac Output | 12.32  (1.66) | 15.94  (0.68) | 19.70  (1.58) | 14.18  (1.42) |
| LV Mass | 90.69  (8.74) | 87.65  (8.52) | 169.02  (17.88) | 116.33  (4.80) |
| LV Mass Cor | 72.55  (6.99) | 70.12  (6.81) | 135.22  (14.31) | 93.06  (3.84) |
